# Supplementary material for: Sparse representation learning derives biological features with explicit gene weights from the Allen Mouse Brain Atlas
Source: PLoS One. 2023 Mar 6;18(3):e0282171. doi: 10.1371/journal.pone.0282171 (PMC9987823; doi:10.1371/journal.pone.0282171)
Supplement: Attachment — Submitted filename: Response to reviewers.pdf [file pone.0282171.s001.pdf]

We thank the reviewers for their insightful comments. We have taken each into account for this manuscript and it has given us food for thought as we continue developing sparse learning methods on AMBA and other datasets. Sections of the text marked in blue denote additions to the manuscript, including methods for new suggested analyses.

We have expanded the manuscript to provide more background and edited where requested to improve the narrative flow. Specifically, we have added a new section to the results to more gradually introduce the concept of interpreting learned weight matrices as signature gene lists, and 4 new paragraphs to the discussion section to better interpret the results and point to future applications. We have also expanded descriptions of the learning tools used in our introduction and methods section to make the methods more accessible to a broader audience.

To address specific comments, please see below.

Visual clustered representations in the area of the olfactory bulb and nucleus will be less accurate in these regions due to registration issues. The brains were distorted somewhat in these areas through processing. Please, clarify what do you mean by a single coronal slice as referred to on line 144.

You will find a clarification between pg 7 and 8. It was important to note these artifacts as they were similar to those discussed in Ortiz 2020, where they were manually curated from their implementation of ICA.

Application of these methods to sagittal oriented sections may be problematic due to the limited resolution in this orientation.

We have included this caveat in the same section and note that our analysis was performed on the volumetric data of AMBA. While the reported resolution of the dataset is 200µm isotropic, the data quality mismatch between sagittal and coronal collected data is likely the root of most artifacts. Much of the data was intractable outside of the set originally curated by Ng and colleagues.

What precisely is the difference in results of SVD versus PCA methods here, as the former is essentially equivalent at full rank. What anatomical features differ?

On page 9 we clarify our use of PCA as equivalent to SVD and that we used the same number of components to recapitulate the Bohland 2010 analysis. We use the term PCA only because it is more familiar to a broad audience.

Please, consider providing a basic explanation of how the application of sparse representation learning algorithms work in this application. References to advances in applied information theory should be given.

On page 11 we provide this explanation and relate our findings back to the major points of this introduction. Several references have been added including text book references to ridge (l2) and LASSO (L1) regression.

Long sections titles such as “Variance based…”on line 151 are overly descriptive without the readers ability to parse meaning. A more intuitive section title with explaining the result early in the first paragraph would be a little easier on the reader.

We have changed the section headings to make them more accessible to a general audience.

Have unsupervised representation learning methods never been applied to or studied for the AMBA dataset? If so, why is that the case and how does this paper address it?

On page 16 we introduce a previous analysis using sparse learning, compare it to other methods, and demonstrate how the reported optimal parameters, while correct for their published analysis, present a surprising artifact in that 95% of the derived features represented the same element of anatomy. This result provides a useful insight into how to best implement sparse learning and contributes to the value of the paper.

It seems the hyperparameters for different algorithms have been selected using the performance on ground truth labels. How will these be selected for other datasets? One experiment could be to split the existing dataset into different sets and see if the hyperparameters selected for one set generalize to another. It is important to answer how can SFt (the proposed method) generalize to other similar datasets and be useful for the community?

On page 26 we discuss how the compressed gene list derived from this analysis could be applied to biology that lacks ground truth labels. We achieved compression by eliminating low rank genes and repeating SFt, essentially splitting the dataset in an informed way.

How do the applied methods in the paper help fix the drawback "Initial dimensionality reduction filters for high variance global trends over localized features"?

On page 4 and 11 we cite how constraining our learning for sparse signatures differs from variance based methods.

" AMI scores peaked well below the 574 labeled brain regions, with most fitting optimally at ~200 clusters" - what does it imply for the applicability of these methods in the domain?

On page 6 and 25 we discuss how resolution limits identification of many smaller brain regions and how the strong signatures can occlude more subtle features. Our major point stands in that learning methods are necessary for complex datasets and for transcriptomic data at least, sparsity constraints return a better fit to ground truth.

How is the feature selection done using the sparse methods affected by the correlation structures in the data? Would these methods suffer from identifiability issues due to highly correlated genes?

On page 25 we further discuss this intriguing question. In our experience gene weights can change drastically across rounds of learning even without changing the data. Our approach to compression was based on this observation where genes were systematically eliminated, then learning was performed.

For data compression, how was the threshold selected to filter out the genes at each step? Why was the SFt step run multiple times? Was the original gene list analyzed with different thresholding?

On page 19 and 20 we have expanded our results section to introduce this idea better and explain the approach. Without repeating learning, removed values would be considered as real 0 values in the previous model. It was necessary to build a new learning model at each step to generate a new matrix. Using the same number of clusters was also necessary for a valid comparison across steps.

There are several optimizations suggested by these results that we are now following up on to develop better informed approaches to gene compression. Those results are necessarily application focused and beyond the scope of this paper.

What is the significance of the other genes reported in the list of 12 highest weighted genes that are not associated with the brain region (highlighted in orange)? For example, what is the role of Pip5k1a that appears for 2 different regions?

On page 25 we discuss how expression levels within a feature can differ from informativeness of expression and present Dock10 as an example. This gene is a marker of CA2, but is highly weighted in each presented representation. Marker genes in general were highly weighted, even when their expression was adjacent to the learned features. We explicitly state feature learning is not a substitute for differential expression analysis.

Table 1 could be better formatted and maybe have the relevant genes in bold text.

We have bolded marker genes for their respective anatomy.

Some figures have empty white spaces on the sides.

We have reformatted figures and legends for this new draft.

Does the logistic regression task have any class label imbalance? If so, AUPRC score might be worth observing as well.

On page 21 we have included AUPRC scores, which also perform well. We have included their generation in the methods section on page 31.

We attempted to address all other comments to the best of our ability and we once again thank the reviewers for their time. We appreciate the opportunity to make this the best manuscript it can be and we are excited to build on our findings.

Thank you.

Benjamin Bartelle PhD
Arizona State University
